# A novel subtype classification for acute intracranial atherosclerotic disease-related occlusion

**DOI:** 10.3389/fneur.2025.1632156

**Published:** 2025-10-07

**Authors:** Shujuan Gan, Tingyu Yi, Meihua Wu, Weifeng Huang, Yi Sui, Yanmin Wu, Shuyi Liu, Zhongrong Miao, Wenhuo Chen

**Affiliations:** 1 Department of Neurology, Fujian Medical University Union Hospital, Fuzhou, China; 2 Interventional Neuroradiology, Department of Neurology, Beijing Tiantan Hospital, Capital Medical University, Beijing, China; 3 Cerebrovascular and Neuro-Intervention Department, Zhangzhou Affiliated Hospital of Fujian Medical University, Fujian, China; 4 Department of Neurology, Shenyang First People’s Hospital, Shenyang Medical College, Shenyang, China

**Keywords:** stroke, intracranial atherosclerotic disease, endovascualar treatment, thrombus, stenosis

## Abstract

**Background and purposed:**

The optimal endovascular therapy (EVT) strategy for intracranial atherosclerotic disease (ICAD)-related occlusion remains uncertain and may be influenced by its underlying pathogenesis. To address this, a novel classification system named Thrombus-Stenosis (TS) has been proposed. Our study aimed to assess the feasibility of the TS classification and its utility in guiding EVT strategy-making.

**Methods:**

ICAD was defined as a significant fixed focal stenosis at the site of occlusion evidenced by final angiography or during endovascular treatment. The TS subtype was classified based on presence of the thrombus and stenosis degree of culprit artery with three categories, TS-type I (<70% stenosis with definite thrombus), TS-type II (≥70% stenosis with definite thrombus) and TS-type III (≥70%stenosis without definite thrombus). Four independent raters used the TS classification system to subtype the included cases. Interobserver reliability was assessed using the kappa (*κ*) coefficient. Differences in EVT strategies between the three TS groups were compared.

**Results:**

A total of 105 definite ICAD-related occlusion cases were included and successfully classified into the three TS subtypes by the four independent raters, with high interobserver agreement (*κ* = 0.95): 33 patients with TS-type I, 46 with TS-type II, and 26 with TS-type III. Compared with TS-type I, the likelihood of performing emergent angioplasty was 44 times higher in TS-type III (95% CI, 5.1–369.8, *p* = 0.001) and 9 times higher in TS-type II (95% CI, 1.1–73.3, *p* = 0.047).

**Conclusion:**

The TS classification system is feasible for subtyping ICAD-related occlusions and is closely associated with EVT strategy-making.

## Introduction

Endovascular therapy (EVT) has become a standard treatment for acute ischemic stroke caused by intracranial large vessel occlusion (LVO) (1). Embolism and intracranial atherosclerotic disease (ICAD) are two major causes of LVO (2). While embolism is common in Western countries, ICAD is prevalent in Asian countries, particularly in China (3). Acute ICAD-related LVO ranges from 40 to 46.6% of cases in China (4). Compared to embolic LVO, ICAD-LVO often requires different technical approaches due to its unique characteristics.

ICAD cases exhibit some unique feature, first, re-occlusion during the acute phase even intraprocedural is common in ICAD case (5, 6). Second, rescue therapies, such as emergent angioplasty with or without stenting, are frequently required (7, 8). The rate of emergent angioplasty with or without stenting range from 32.4% (9) to 81.7% (10).

To date, the optimal EVT strategy for ICAD remains uncertain. Neuro- interventionists may face several key questions when making EVT decisions in ICAD cases: first, whether to perform retrieval-stent thrombectomy; second, whether to perform emergent angioplasty; and third, when to perform emergent angioplasty. To address these dilemmas, two key factors should be considered: the presence of a thrombus at the occlusion site, which determines whether to perform clot retrieval (11) and the degree of stenosis at the culprit site, which determines whether to perform angioplasty (12). Therefore, we propose a classification system Thrombus-Stenosis (TS), that focuses on these two critical factors to help neuro-interventionists develop appropriate EVT strategies.

This study was designed to assess the feasibility of the TS classification in subtyping acute ICAD-related occlusion, evaluate its practicality in guiding EVT strategy decisions for acute ICAD-related occlusion, and investigate the differences in baseline clinical features and periprocedural imaging among the different TS subtype groups.

## Methods

This study is based on a prospective, hospital-based registry involving consecutive patients with large vessel occlusion (LVO) who were treated with endovascular therapy (EVT). Upon admission to the hospital, detailed information regarding patient demographics, medical and endovascular interventions, periprocedural management approaches, and clinical outcomes was systematically collected and entered an electronic database. Informed consent was obtained from all patients or their legally authorized representatives prior to the commencement of EVT. Additionally, the study protocol received approval from local institutional review board.

### Inclusion and exclusion criteria

The inclusion criteria were as follows: (1) acute ischemic stroke (AIS) caused by intracranial large artery occlusion (LVO), include intracranial carotid artery (ICA), middle cerebral artery (MCA); (2) etiology of LVO was intracranial atherosclerotic disease (ICAD); (3) the onset-to-presentation time was within 24 h; (4) underwent endovascular treatment (EVT) and achieved successful recanalization that can stenosis degree of culprit artery; (5)aged 18 years or older; and (6) the pre-stroke modified Rankin Scale (mRS) score was between 0 and 2.

The exclusion criteria were as follows: (1) The cause of LVO was cardioembolic, dissection, moyamoya disease, vasculitis, or undetermined; (2) posterior circulation LVO; (3) tandem occlusion; (4) distal medium artery occlusion; (5) underwent stenting; (6) repeated intraprocedural re-occlusion or early re-occlusion post procedure; (7) lack of high-resolution magnetic resonance imaging (HR-MRI); (8) poor imaging included DSA/CTA/MRA quality to analysis; (9) absence of 90-day follow-up outcome data.

### Definitions of ICAD

ICAD was defined as a significant fixed focal stenosis at the site of occlusion evidenced by final angiography or during endovascular treatment (3). Arteriosclerotic plaques were observed at culprit artery on high-resolution MRI performed after procedure.

### Endovascular procedures

Thrombectomy with retrieval stent or/and aspiration catheter was determined by neuro-interventionist. All treatment decisions during EVT, including emergent angioplasty, were made in real-time based on standard angiographic findings and were not influenced by the TS classification since this was a retrospective study. Performing emergent angioplasty was more depended on reperfusion status rather than residual stenosis degreee (13). It was performed only when high-grade residual stenosis co-existed with adequate antiplatelet coverage and at least one of the following conditions: (1) impaired distal flow (eTICI grade ≤ 2a) after mechanical thrombectomy; (2) re-occlusion or significant flow impared during the procedure; and (3) angiographic evidence of high re-occlusion risk (e.g., significant residual stenosis with delayed antegrade flow or poor collateral compensation).

### TS subtype classification system

The TS classification system was determined based on DSA images, with two aspects of the images being considered, namely, the existence of thrombus and the residual stenosis degree. The residual stenosis degree was measured in the absence of vasospasm or dissection appearance or residual thrombus after successful recanalization achieved by mechanical thrombectomy. Cases with dissection appearance or residual thrombus or vasospasm on DSA imaging were excluded from this study. The degree of stenosis was assessed using the Warfarin–Aspirin Symptomatic Intracranial Disease Study (WASID) method. According to this method, the degree of stenosis was classified as mild to moderate (<70%) or severe (70–99%) (14). The presence of a definite thrombus was defined in the following two ways: (1) Macroscopic thrombus visible on the extracted stent- retriever or aspiration catheter which was documented in medical record; and/or (2) distal embolism within the target vessel territory after a thrombectomy pass, as recorded in the procedural note and confirmed by subsequent DSA. The degree of stenosis was semi-automatically quantified using the built-in measurement tools on the DSA workstation.

**Figure 1:**
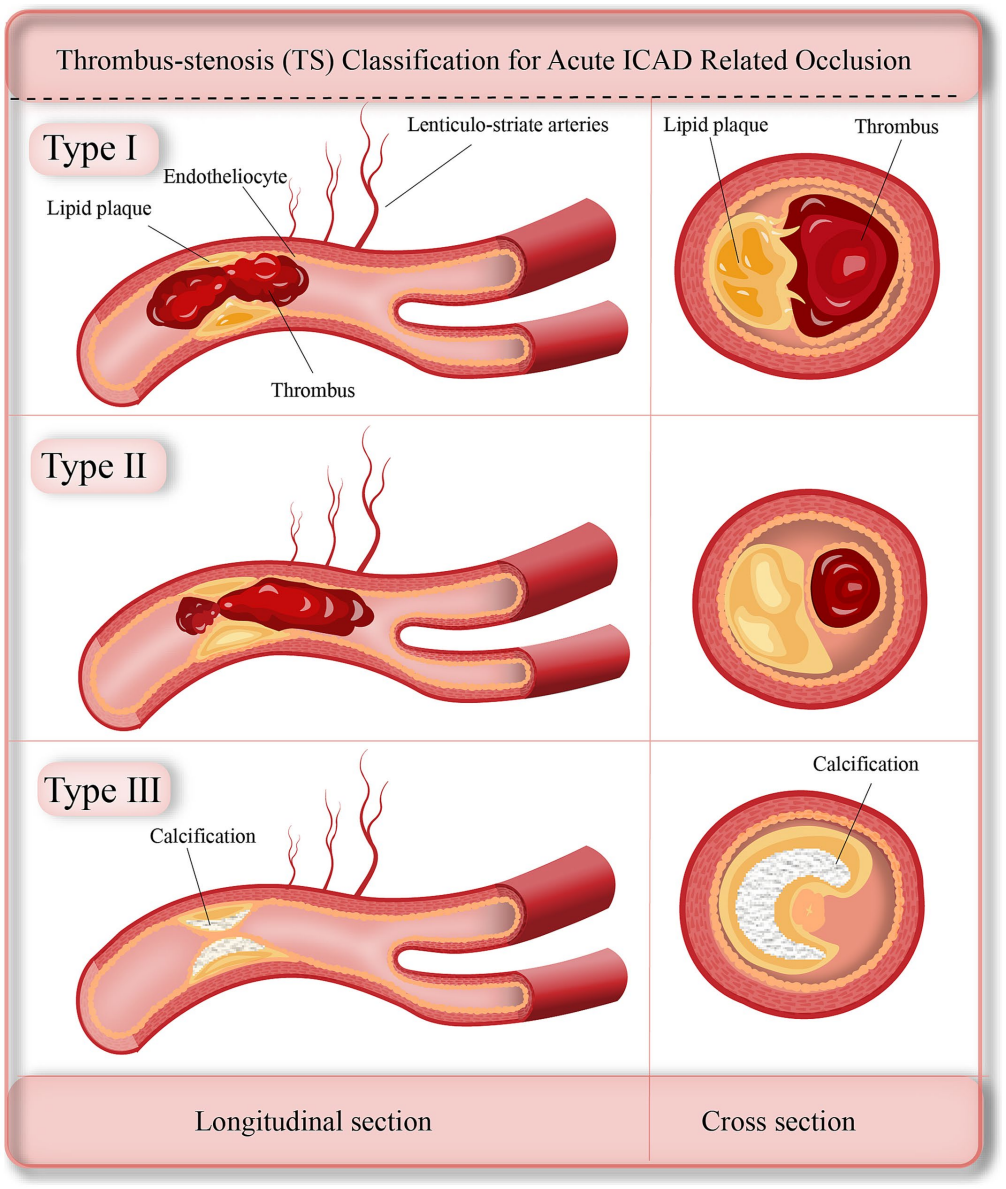
Schematic illustration of the TS classification system. Type I: mild-to-moderate stenosis with thrombus; Type II: severe stenosis with thrombus; Type III: severe stenosis without thrombus.

The TS classification consists of three types (Figure 1):

TS - type I: residual stenosis rate <70% with a definite thrombus;
TS - type II: residual stenosis rate≥70% with a thrombus;
TS - type III: residual stenosis rate≥70% without a thrombus.

The TS classification was determined by two independent neurologists, Dr. Pan and Dr. Lin, who had not participated in the writing of the criteria (group A), and two independent neuro-interventionists, Dr. A and Dr. B, who had participated in the writing of the criteria (group B). In cases of inter-observer disagreements, a consensus was reached to make the final determination. Dr. C is an attending neurologist with 7 years of experience in neurointerventional surgery. Dr. D is a resident neurologist with 6 years of experience in neurology and 3 years of experience in neurointerventional surgery. Dr. A and B are consulting neuro-interventionists, each having performed more than 500 operations per year.

### Evaluation of angiographic and clinical outcome

All angiographic classifications, including the residual stenosis degree and the reperfusion score, were evaluated by the two independent raters (Z. N. P. and D. L. L.). Interobserver disagreements were resolved by consensus. The reperfusion statuses were measured using the extended Thrombolysis in Cerebral Infarction (eTICI) scale (15). An eTICI of 2b-3 was classified as successful reperfusion (15). Intraprocedural re-occlusion rate was high in ICAD-related LVO (16), so thrombectomy reperfusion should be observed at less 20 min during procedure (13). The first pass effect (FPE) is defined as the achievement of complete reperfusion (eTICI 3) after a single pass of thrombectomy (17).

Occlusion length was measured base on the venous phase of multi-phase CTA imaging (18). Perfusion maps and ischemic core volumes (CTP volumes) were auto-calculated by MIStar software (Apollo Medical Imaging Inc., Melbourne, VIC, Australia) (19). Delayed time (DT > 3 s) was defined as hypoperfusion brain tissue, while the ischemic core volumes were defined by relative cerebral blood flow (rCBF) < 30%. The mismatch volume was calculated as the volume of DT > 3 s minus the volume of rCBF < 30%, and the mismatch ratio was determined by dividing the volume of DT > 3 s by the volume of rCBF < 30%.

Clinical outcome was assessed by 3-month’s mRS, and favorable outcome was defined as mRS of 0–2, excellent outcome as mRS 0–1, and symptomatic intracranial hemorrhage (sICH), defined as any type of hemorrhage associated with an increase in the NIHSS score by ≥4 points (20).

### Statistical analysis

Patients were categorized into three groups based on the TS classification criteria by four independent readers. In cases of disagreement, the relevant cases were re-examined, and any disputes were resolved through consensus. The interobserver reliability was assessed using the kappa (k) coefficient, with k values ranging from 0.70 to 0.89 indicating good consistency, and values between 0.9 and 1 representing excellent consistency. Quantitative variables were presented as the mean and standard deviation (SD) or the median and interquartile range, while categorical variables were expressed as the number of cases (N) and frequencies (%). For continuous variables with a normal distribution, Student’s *t*-test was conducted, whereas the Mann–Whitney *U*-test was utilized for continuous variables without a normal distribution or ordinal variables. Analysis of variance (ANOVA) was performed for comparisons among the three groups, followed by Bonferroni correction for pairwise comparisons. The chi-square (*χ*^2^) test was applied to categorical variables. Variables with a *p* < 0.05 were incorporated into the multivariable regression model to ascertain the correction between TS Subtype with EVT strategy and clinical Outcome, with odds ratios (ORs) being calculated. All statistical analyses were executed using SPSS 20.0 (IBM, Armonk, New York, USA) and R statistical software (Version 4.1.3), and a *p* < 0.05 was deemed statistically significant.

### Data

Anonymized data will be shared upon request to any qualified investigator.

## Results

### Study population

**Figure 2:**
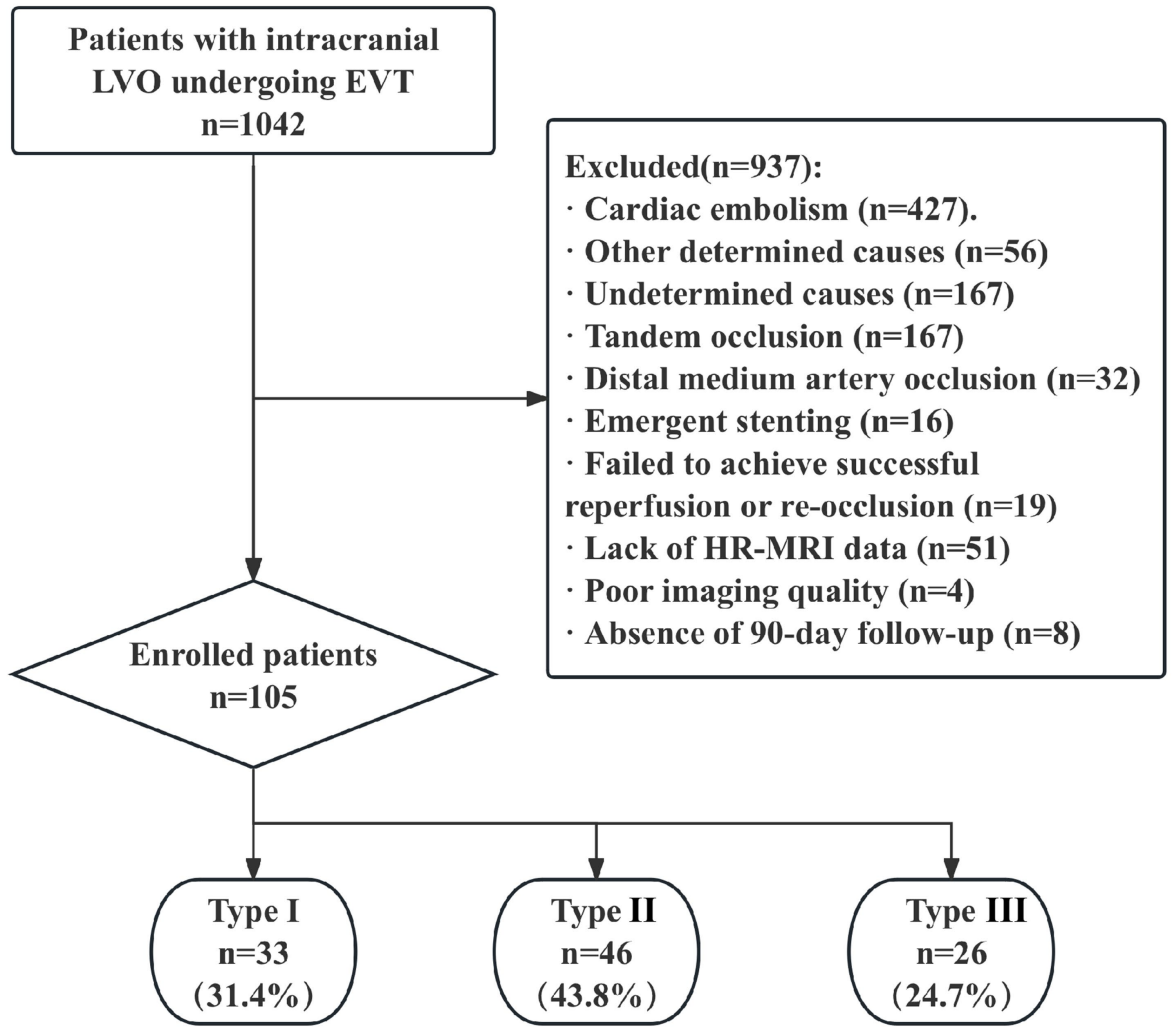
Study flow chart. AIS, acute ischemic stroke; EVT, endovascular therapy; LAA, large-artery atherosclerosis; ICA, intracranial carotid artery; MCA, middle cerebral artery; ICAD, intracranial atherosclerotic disease; HR-MRI, high resolution MRI.

Between January 2020 and December 2022, a total of 1,042 patients with intracranial LVO who underwent EVT were initially included in this study (Figure 2). After excluding patients for the following reasons, 105 patients (mean age 61.9 ± 10.5 years; male: female ratio 87:18) were finally enrolled: (1) cardiac embolism (*n =* 427), other determined causes (*n =* 56), and undetermined causes (*n =* 197); (2) tandem occlusion (*n =* 167); (3) distal medium artery occlusion (*n =* 32); (4) emergent stenting (*n =* 16); (5) failed to achieve successful reperfusion or re-occlusion during procedure or early after procedure (*n =* 19); (6) lack of high-resolution magnetic resonance imaging (HR-MRI) data (*n =* 51); (7) poor imaging quality (*n =* 4); and (8) absence of 90-day follow-up outcome data (*n =* 8).

### Testing TS classification

The included patients were independently evaluated by two neurologists who had not participated and two neuro-interventionists who were involved in the development of the TS subtype diagnosis system. Prior to patient assessment, the four raters reached a consensus to strictly adhere to the classification system’s criteria without any exceptions. The thrombus retrieved by the device was physically documented and traceable in each patient’s record, while the degree of stenosis was semi-automatically quantified using the built-in measurement tools on the DSA workstation. So, excellent inter-observer agreement for TS classification was achieved in both individual groups (A and B) and across the combined cohort, with κ = 0.99. Among the 105 patients, the four raters concurred on the final diagnosis of the subtypes, except for one patient. This patient had a residual stenosis of less than 70% and no thrombus trapped by the stent. It was speculated that the thrombus could have been dissolved when the stent was unsheathed and intravenous tirofiban was administered. Consequently, this patient was classified as TS - type I.

### Comparison of baseline characteristic among the three TS subtypes

**Figure 3:**
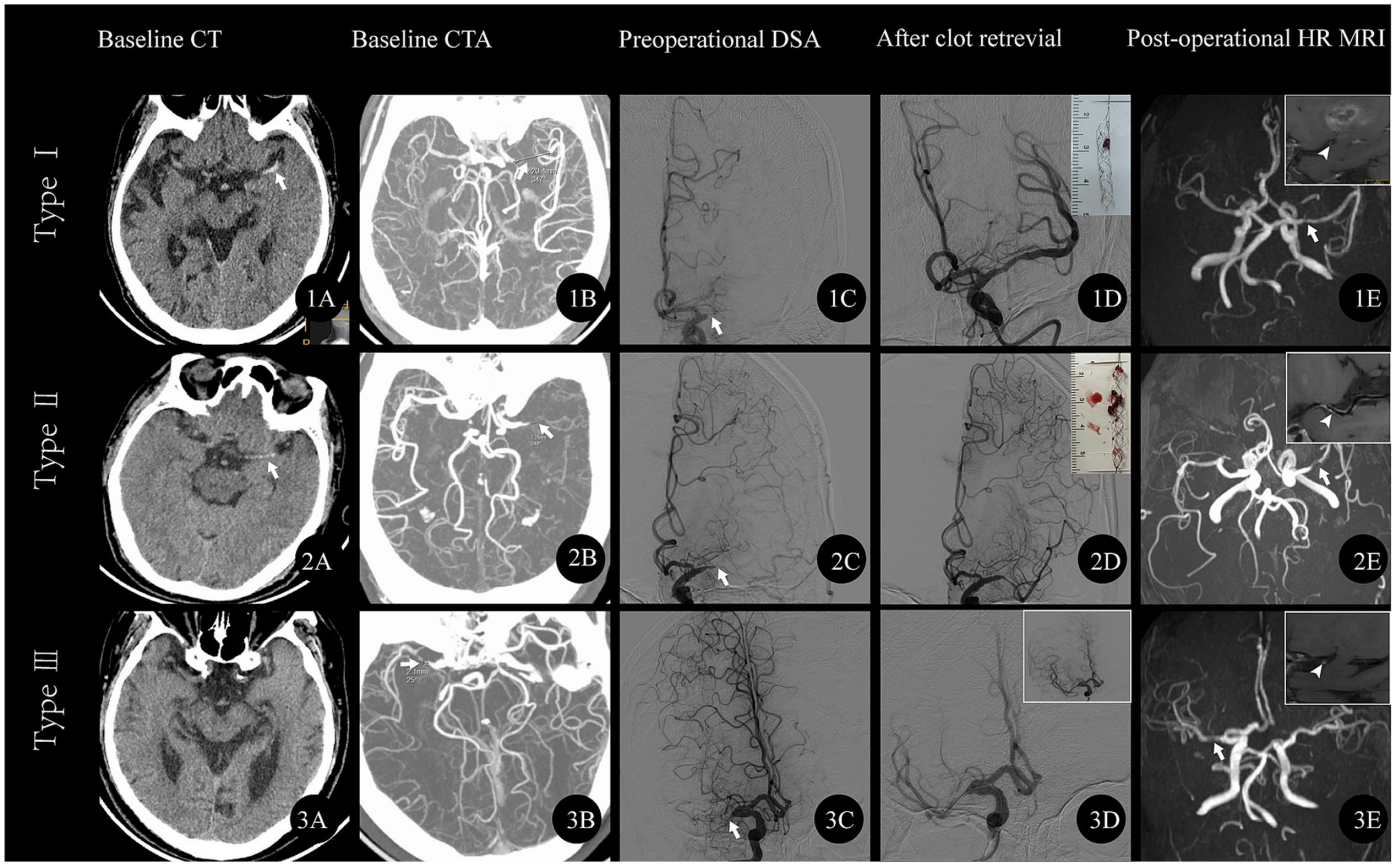
Representative cases of respective TS subtype. **(A)** TS-type I A middle-aged male patient presented with sudden right limb weakness lasting 19 h, muscle strength grade 0, left eye deviation, and NIHSS score of 17. **(1A)** Baseline CT shows HAS in the left MCA (arrow); **(1B)** CTA reveals left MCA occlusion with an occlusion length of 14 mm; **(1C)** Angiogram confirms left MCA occlusion (arrow); **(1D)** Post-clot retrieval angiogram shows mild stenosis in the left MCA; the thrombus is visualized in the upper right frame; **(1E)** Follow-up MRA shows mild stenosis in the left MCA (arrow). The high-resolution MR (TIWI) image in the upper right frame reveals an irregular plaque within the left MCA (white arrowhead). **(B)** TS-type II A middle-aged male patient presented with sudden right limb weakness lasting 8 h, muscle strength grade 4, and NIHSS score of 3. **(2A)** Baseline CT shows HAS in the left MCA (arrow); **(2B)** CTA reveals left MCA occlusion with an occlusion length of 7.2 mm; **(2C)** Angiogram confirms left MCA occlusion (arrow); **(2D)** Post-clot retrieval angiogram shows severe stenosis in the left MCA; the thrombus is visualized in the upper right frame; **(2E)** Follow-up MRA shows severe stenosis in the left MCA (arrow). The high-resolution MR (TIWI) image in the upper right frame reveals an irregular plaque within the left MCA (white arrowhead). **(C)** TS-type III An elderly patient presented with sudden left limb weakness lasting 2 h, muscle strength grade 4, and NIHSS score of 5. **(3A)** Baseline CT is normal; **(3B)** CTA reveals right MCA occlusion with an occlusion length of 2.1 mm; **(3C)** Angiogram confirms right MCA occlusion (arrow); **(3D)** Post-clot retrieval angiogram shows severe stenosis in the right MCA without thrombus. A subsequent angiogram (upper right frame) shows mild stenosis in the right MCA; **(3E)** Follow-up MRA shows mild stenosis in the right MCA (arrow). The high-resolution MR (TIWI) image in the upper right frame reveals an irregular plaque. NIHSS, National Institutes of Health Stroke Scale; HAS, hyperdense artery sign; MCA, middle cerebral artery; CTA, CT angiography; MRA, Magnetic Resonance Angiography.

Based on the TS classification criteria, 31.4% (33/105) of patients were classified as TS-type I (a representative case is depicted in Figure 3A), 43.8% (46/105) as TS-type II (Figure 3B), and 24.7% (26/105) as TS-type III (Figure 3C).

The baseline characteristics are presented in Table 1. The TS-type I group exhibited a larger baseline infarct core volume (11 mL vs. 3 mL vs. 4 mL, *p* = 0.008) compared to the other two groups. The TS-type III group had a shorter occlusion length (7.4 mm vs. 7.7 mm vs. 3.5 mm, *p* < 0.001) and a lower prevalence of HAS (24.2% vs. 32.6% vs. 0%, *p* = 0.015) than the other subtypes.

**Table 1:** Baseline clinical features and imaging characteristics among different TS subtype groups.

Parameters	Total (*n =* 105)	Type I (*n =* 33)	Type II (*n =* 46)	Type III (*n =* 26)	*P*
Age, year, (mean ± SD)	61.9 ± 10.5	60.6 ± 9.7	61.33 ± 10.4	64.4 ± 11.4	0.348
Male sex, n (%)	87 (82.9)	30 (90.9)	38 (82.6)	19 (73.1)	0.196
NIHSS, median (IQR)	9 (6–12.5)	10 (7–14)	8.5 (6–12)	8 (6–12)	0.416
ASPECTS, median (IQR)	9 (7–10)	8 (7–10)	9 (7–10)	9 (8–10)	0.672
SBP mmHg (mean ± SD)	152.7 ± 26.6	146.6 ± 27.5	157.6 ± 26.5	151.8 ± 24.8	0.191
Progressive stroke, n (%)	26 (24.8%)	9 (27.3)	10 (21.7)	7 (26.9)	0.818
Vascular risk factors, n (%)					
Smoking	27 (25.7)	8 (24.2)	12 (26.1)	7 (26.9)	0.970
Hypertension	74 (70.5)	20 (60.6)	32 (69.6)	22 (84.6)	0.131
Diabetes	33 (31.4)	8 (24.2)	18 (39.1)	7 (26.9)	0.316
Hyperlipidemia	17 (16.2)	5 (15.2)	7 (15.2)	5 (19.2)	0.889
Prior stroke or TIA	12 (11.4)	4 (12.1)	5 (10.9)	3 (11.5)	1.000
Intravenous rt-PA, *n* (%)	15 (14.3)	6 (18.2)	7 (15.2)	2 (7.7)	0.525
Image Characteristics					
HAS *n* (%)	23 (21.9)	8 (24.2)	15 (32.6)	(0)	0.005
Length of occlusion, median (IQR)	6 (4.25–9.15)	7.36 (5.4–9.32)	7.64 (5.5–10.1)	3.5 (2.3–5.2)	<0.001
CTP parameters, median (IQR)					
DT > 3 s	85.5 (52–118.5)	90 (53–115)	80.5 (48–108.8)	96.8 (53–137)	0.440
rCBF<30%	5 (1–15)	11 (4–24)	3 (1–10)	4 (1–8)	0.008
Mismatch	73 (48.1–105)	76 (50–99)	71.5 (47–97)	81 (49–123)	0.576
Workflow, minutes, median (IQR)					
ODT	433 (188–995.5)	472 (209–745)	432.5 (169–1,089)	366 (182–1,205)	0.956
DPT	99 (83–160.5)	98 (85–118)	114 (82–182)	95.5 (86–137)	0.364
ORT	641 (384–1242.5)	618 (350–969)	645.5 (347–1,599)	623 (405–1,464)	0.442^‡^
PRT	54 (42–68)	54 (45–61)	57 (40–72)	49.5 (38–86)	0.698^‡^

SD, standard deviation; IQR, interquartile range; NIHSS, National Institutes of Health Stroke Scale; ASPECTS, Alberta Stroke Program Early CT Score; SBP, systolic blood pressure; TIA, transient ischemic attack; rt-PA: recombined tissue plasminogen activator; HAS, hyperdense artery sign; CTP: CT perfusion; DT, delayed time; rCBF, relative cerebral blood flow; ODT, onset to door time; DPT: door to puncture time; ORT: onset to reperfusion times; PRT, puncture to reperfusion time.

### Comparison of radiological and clinical outcomes among the three TS subtypes

The radiological and clinical outcomes are summarized in Table 2. Compared to the other subtypes, TS-type III patients more frequently underwent emergent angioplasty (0% vs. 19.6% vs. 53.8%, p < 0.001) and had a lower rate of FPE (90.9% vs. 63.0% vs. 34.6%, *p* < 0.001). TS-type II patients were less likely to achieve eTICI 2c-3 reperfusion (97.0% vs. 82.2% vs. 100%, *p* = 0.017), required a higher number of thrombectomy passes (1.0 vs. 1.5 vs. 1.0, *p* = 0.003), needed more pass of thrombectomy (≥3 passes; 0% vs. 13% vs.0%, *p* = 0.021).

**Table 2:** EVT details, radiological and clinical outcome among different TS subtype groups.

Parameters	Total (*n =* 105)	Type I (*n =* 33)	Type II (*n =* 46)	Type III (*n =* 26)	*P*
EVT details					
Angioplasty, *n* (%)	31 (29.5)	0 (0)	9 (19.6)	14 (53.8)	<0.001
Pass of thrombectomy, mean±SD	1.22 ± 0.6	1.03 ± 0.2	1.46 ± 0.8	1.04 ± 0.4^a^	0.003
Pass of thrombectomy, *n* (%)					0.021
None	2 (1.9)	0 (0)	0 (0)	2 (1.9)	
One pass	86 (81.9)	32 (97.0)	33 (71.7)	21 (80.8)	
Two passes	11 (10.5)	1 (3.0)	7 (15.2)	3 (11.5)	
Three passe	4 (3.8)	0 (0)	4 (8.7)	0 (0)	
Four passes	2 (1.9)	0 (0%)	2 (4.3)	0 (0)	
Radiological outcomes, *n* (%)					
eTICI 2c-3	96 (91.4)	32 (97)	37 (82.2)	27 (100)	0.017
FPE	68 (64.8)	30 (90.9)	29 (63.0)	9 (34.6)	<0.001
DE	6 (5.7)	4 (12.1)	2 (4.3)	2 (0)	0.139
sICH	0	0	0	0	1.00
H1/H2	5 (4.8)	2 (6.1)	0 (0)	3 (11.5)	0.055
SAH	5 (4.8)	1 (3)	3 (6.5)	1 (3.8)	0.853
PH1	1 (1.0)	0 (0)	1 (2.2)	0 (0)	1.000
Clinical outcomes, *n* (%)					
mRS 0–1 at 90 days	79 (75.2)	25 (75.8)	35 (77.8)	19 (70.4)	0.777
mRS 0–2 at 90 days	91 (86.7)	28 (84.8)	39 (86.7)	24 (88.9)	0.936

EVT, endovascular therapy; SD, standard deviation; eTICI, extended thrombolysis in cerebral infarction; FPE, first pass effect; DE, distal embolism; sICH, symptomatic intracranial hemorrhage; SAH, subarachnoid hemorrhage; PH1, parenchymatous hematoma; mRS, modified Rankin Scale.

### Relationship between TS subtypes and EVT strategy

When compared with TS-type I patients, the likelihood of performing emergent angioplasty was significantly higher in TS-type III patients (aOR,44.0; 95% CI,5.1–369.8; p < 0.001) and TS-type II patients (aOR, 9.0; 95% CI,1.1–73.3; *p* = 0.047), after adjustment for age and NIHSS (Table 3).

**Table 3:** Correlation of TS subtype with EVT strategy and clinical outcome.

EVT parameters, *n* (%)	I	II aOR (95%CI)	*P*	III aOR (95%CI)	*P*
Angioplasty	Reference	9.0 (1.1–73.3)	0.047	44.0 (5.1–369.8)	0.001
Outcomes, *n* (%)					
mRS 0–1 at 90 days	Reference	1.0 (0.3–3.0)	0.977	0.9 (0.2–3.1)	0.841
mRS 0–2 at 90 days	Reference	1.3 (0.3–5.0)	0.841	1.6 (0.3–8.6)	0.56

EVT, endovascular therapy; aOR, adjusted odds ratio; CI, confidence interval. Model analysis adjusted by age and baseline NIHSS.

## Discussion

To our knowledge, our study is the first to introduce the ICAD-related occlusion classification, namely the TS subtype, which was defined based on DSA imaging data obtained during EVT. The TS classification system is user-friendly and sufficiently practical for physicians in clinical practice. Moreover, the system is simple and clear, and our data indicate a high level of interrater agreement in diagnosing ICAD-related occlusion subtypes when physicians apply the TS classification system to individual cases.

The TS subtype classification is based on the presence of thrombus and the degree of residual stenosis, which are key factors that neuro-interventionists consider when making EVT strategies. Severe intracranial artery stenosis, defined as ≥70%, serves as the criterion for angioplasty (21). In the TS subtype classification, we adopted a stenosis degree of ≥70% as indicative of severe stenosis, which guides neuro-interventionists in deciding whether to perform angioplasty. The presence or absence of thrombus is related to the decision of whether to perform clot retrieval (22).

Mathematically and logically, these two factors can generate four combined modes, namely ≥70% with or without thrombus and <70% with or without thrombus. However, the likelihood of culprit artery occlusion is very low if the stenosis degree of the culprit artery is mild-to-moderate and there is no thrombus. Therefore, there are only three subtypes in our TS classification system. All the included ICAD cases in our study, except for one, can be classified into the corresponding subtypes. In that specific case, we observed the appearance of thrombus at the culprit site. We retrieved the stent 10 min after the intravenous administration of tirofiban (23), leading us to suspect that the thrombus had resolved when the antegrade blood flow was restored and the anti - platelet agents took effect. Consequently, this case was classified as TS-type I. Thus, we believe that the TS classification system can effectively classify all ICAD cases.

The TS classification represents the first-ever subtype categorization for acute ICAD-related occlusion. To ensure the accuracy of the TS classification, only ICAD cases confirmed by postoperative HR-MRI and patients who achieved successful reperfusion were included in our study. This selection criterion likely accounts for the better prognosis observed in our cohort compared to other studies, as well as the absence of sICH in our patient group (12, 16, 24). It should be noted that ICAD cases involving stenting, which may occur in TS-types II and III, were excluded from our study. This is because patients who had received stenting could not undergo HR-MRI to confirm the ICAD diagnosis. However, in our center, less than 5% of ICAD-related LVO cases received stenting, which minimizes the potential for selection bias in our study.

The TOAST classification is a typical subtype classification of AIS (25), but its pathogenesis does not focus on the stenosis degree and clot burden of the culprit artery. Therefore, the TOAST classification may not be precisely instructive for EVT strategy-making in ICAD-related occlusion cases. The Mori subtype was originally designed to guide EVT strategy-making for intracranial artery stenosis (26), but it is also less instructive for acute ICAD-related occlusion. The Mori classification, focused solely on stenosis morphology and length, and comprise A, B and C three subtypes, overlook any assessment of thrombus burden, which was a critical determinant in selecting the optimal first-line strategy for acute ICAD-related occlusions. In contrast, our TS classification considers both the presence or absence of thrombus and the residual stenosis degree at the occlusion site. This broader consideration provides guidance for the emergent EVT strategy in recanalizing ICAD-related occlusions. In our study, the need for emergent angioplasty was significantly dependent on the severity of residual stenosis, i.e., angioplasty was approximately 5-fold more frequent in TS-type II cases and 25-fold more frequent in TS-type III cases compared to TS-type I. These results enhance the practicability of the TS classification.

In TS-type I cases, approximately 94% of patients achieved successful reperfusion after thrombectomy using a stent retriever or aspiration, suggesting that direct clot retrieval may be an optimal strategy for TS-type I. In contrast, more than 60% of patients with TS-type III underwent emergent angioplasty to achieve successful reperfusion, and no thrombus was observed in any TS-type III cases, indicating that direct emergent angioplasty may be more suitable for TS-type III. The treatment dilemma for TS - type II was the high degree of lesion stenosis, which may restrict the retrieval of clots located distal to the stenotic site. Consequently, TS-type II patients required a higher number of stent retriever passes and had a lower rate of complete reperfusion. In this regard, the BASIS technique, recently proposed by our team, can successfully address the technical difficulties associated with TS-type II (27). All practical EVT strategies in our study aligned with the assumptive EVT strategy for each respective TS subtype. Moreover, higher rates of favorable functional outcomes, absence of sICH and re-occlusion in our study supported the correctness of the strategies we made based on the TS classification. Therefore, thrombectomy randomized controlled trials (RCTs) based on the TS classification are warranted for acute ICAD-related occlusion.

Similar to the pathogenesis of acute coronary syndromes (28), acute ICAD-related occlusion may involve three primary pathogenetic mechanisms: (1) TS-type I is characterized by acute thrombus formation resulting from unstable plaque rupture; and the (2) TS-type II involves the development of secondary red blood cell (RBC)-rich thrombi either proximal or distal to a stenotic site. This occurs due to slow blood flow caused by culprit artery occlusion, often linked to high-grade stenosis with minimal *in situ* formation of fibrin-and platelet-rich thrombi; (3) TS-type III is associated with occlusion related to high-grade stenosis but with little or no local thrombus formation. HAS refers to thrombi rich in red blood cells (29), typically indicative of newly formed thrombi (29). Consistent with the distinct pathogeneses of each TS subtype, the prevalence of HAS was higher in TS-types I and II compared to TS-type III. Additionally, the occluded vessel length was shortest in TS-type III, likely due to the limited thrombus burden and dominant role of severe stenosis in this subtype.

In AIS due to LVO, primary and secondary collateral circulation often activates (30). Chronic focal cerebral ischemia from intracranial artery stenosis can enhance secondary collaterals (30–32), which are linked to smaller infarct cores (33, 34). Our TS classification reflects this: Types II and III have smaller infarct cores than Type I, likely due to better collateral circulation.

The notably high rate of excellent clinical outcomes and the absence of any association between TS classification and outcome in our cohort warrant careful consideration. This discrepancy likely reflects the stringent patient-selection process, in which HR-MRI was mandatory to confirm an ICAD diagnosis and ensure the accuracy of TS categorization. Consequently, our study may have preferentially enrolled patients with inherently favorable prognoses. Prospective investigations are therefore required to re-evaluate the relationship between TS classification, clinical outcome, and response to thrombectomy in a broader, less-selected population.

### Limitations

Our study has several limitations. First, its retrospective single center nature may introduce uncontrolled bias, though prospective data collection helped minimize the influence, in future, multi-center studies are needed to validate the practicability and clinical significance of TS classification. Second, the TS subtype classification, based on EVT results (e.g., thrombus trapped by the stent and residual stenosis), is only applicable to patients who underwent successful EVT recanalization. Future studies will prospectively validate the TS system’s utility in guiding EVT strategies and use larger samples to identify clinical/imaging markers for predicting TS subtypes, thereby informing preoperative EVT decision-making. Third, bridging intravenous thrombolysis may dissolve thrombi at proximal or distal occlusion sites, potentially affecting TS subtype classification. In forthcoming work we would derive a predictive model for TS classification that incorporates clinical presentation and baseline brain imaging, thereby mitigating this limitation. Fourth, the relationship between TS classification and long-term clinical outcomes was not examined in the present study and warrants dedicated investigation in future work.

### Future direction

Once the TS classification has been established, three priority directions emerge for future research: (1) Prospective, interventional trials stratified by TS subtype, for example, head-to-head comparisons of optimal endovascular strategies for Type I versus Type III lesions; (2) Prospective cohort studies examining the association between TS subtype and short and long-term clinical outcomes. (3) Development and validation of predictive models that integrate clinical presentation and baseline brain imaging to pre-procedurally estimate TS subtype.

## Conclusion

In this study, we proposed a new classification system of ICAD-related occlusion, specifically for AIS patients receiving EVT. The TS subtypes are closely related to the volume of infarct cores, achievement of FPE and chance of angioplasty, which are all instructive for neurointerventional strategy. Emergent angioplasty is frequently required in TS subtypes II and III, while clot retrieval is more common in TS subtype I.

## Data Availability

The data analyzed in this study is subject to the following licenses/restrictions: Relating to patient privacy. Requests to access these datasets should be directed to WC, doctorwwenhuo@126.com.
