# Correction: A novel subtype classification for acute intracranial atherosclerotic disease-related occlusion

**DOI:** 10.3389/fneur.2025.1766195

**Published:** 2026-01-08

**Authors:** Shujuan Gan, Tingyu Yi, Meihua Wu, Weifeng Huang, Yi Sui, Yanmin Wu, Shuyi Liu, Zhongrong Miao, Wenhuo Chen

**Affiliations:** 1 Department of Neurology, Fujian Medical University Union Hospital, Fuzhou, China; 2 Interventional Neuroradiology, Department of Neurology, Beijing Tiantan Hospital, Capital Medical University, Beijing, China; 3 Cerebrovascular and Neuro-Intervention Department, Zhangzhou Affiliated Hospital of Fujian Medical University, Fujian, China; 4 Department of Neurology, Shenyang First People's Hospital, Shenyang Medical College, Shenyang, China

**Keywords:** stroke, intracranial atherosclerotic disease, endovascualar treatment, thrombus, stenosis

In the published article, there was a mistake in figure 2. The three subtype boxes at the bottom of the flowchart were incorrectly labeled “Type I” for all three groups. The correct labels should be Type I, Type II, and Type III respectively (with the same group sizes), but the published figure shows “Type I” repeated three times. The corrected Figure 2 appears below.

**Figure 2:**
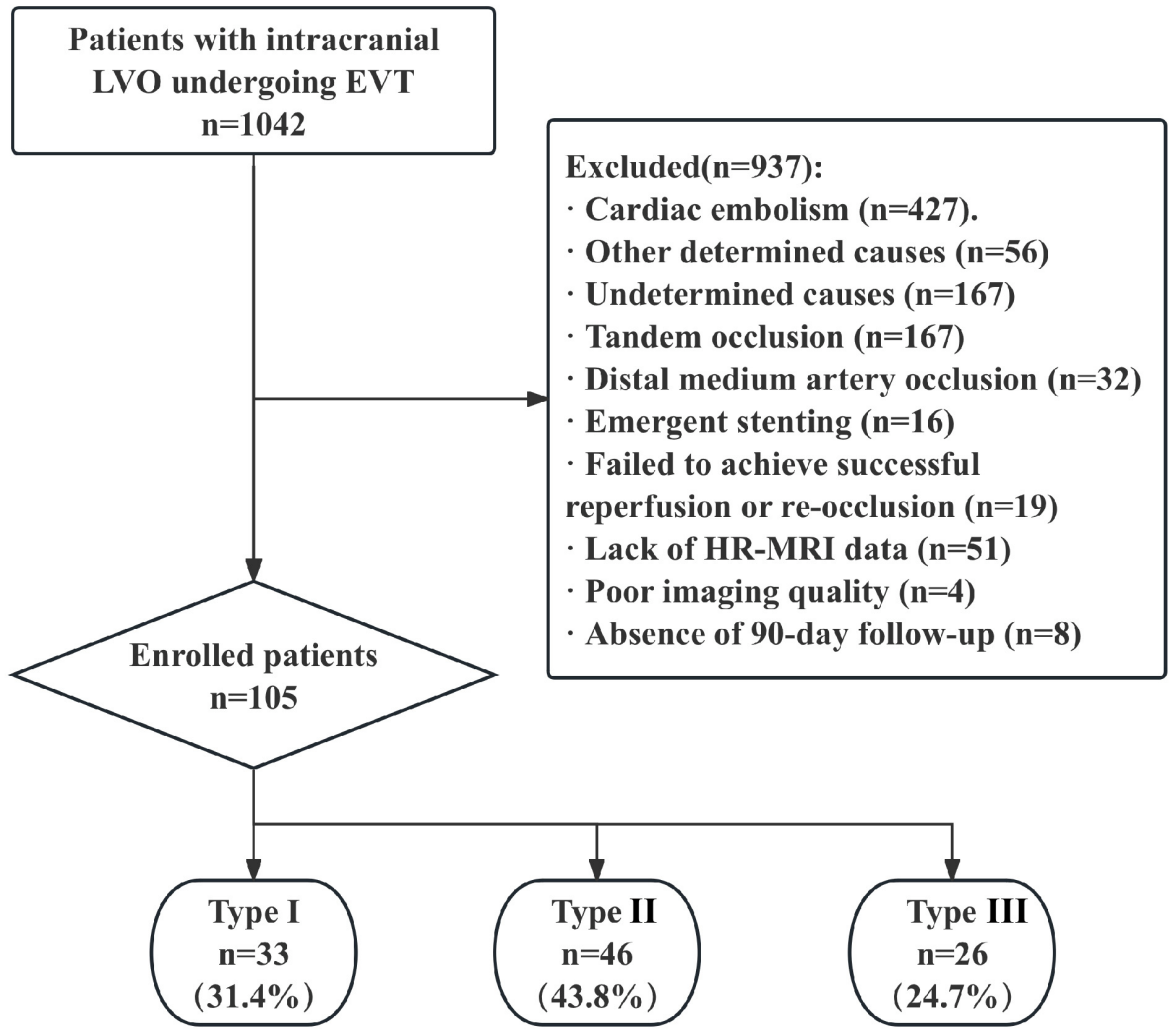
Study flow chart. AIS, acute ischemic stroke; EVT, endovascular therapy; LAA, large-artery atherosclerosis; ICA, intracranial carotid artery; MCA, middle cerebral artery; ICAD, intracranial atherosclerotic disease; HR-MRI, high resolution MRI.

The original version of this article has been updated.

